# 

**Published:** 1873-09

**Authors:** 


### CONTENTS.

ORIGINAL COMMUNICATIONS.

Address Before the Lowndes County (Miss.) Medical Society, at the Anniversary Meeting, May 29, 1873. By Drs. J. W. M. Shattuck and W. L. Lipscomb—Concluded, . . . . . . . . . . . . 513
The Treatment of the Intestinal Disorders of Children. By S. Henry Dessan, M.D., . . . . . . . . . . . . 520
Medical Extracts—Practical and Philosophical. By L. B. Anderson, M.D., 528
Dislocation of the Spleen. By A. R. Kilpatrick, M.D., . . . . . 534
Gastric Hematamesis, Nicarious of Regular Menstruation, Produced by Malarial Causes. By E. M. Vasser, M.D., . . . . . . . 536
The Use of Black Cohosh During Pregnancy. By Ely Van DeWarker, M.D., 539

EXTRACTS AND GLEANINGS.

Fischl on Pneumonia Migrans—541. Treatment of Cystitis; Iodoform for Prurigo and Hemorrhoids—542. The Internal Use of Aconite in Neuralgia; Diabetes—543. Headache in Children; Treatment of Fissure of the Anus—544. Cases of Traumatic Malignant Onychia Successfully Treated by Means of Nitrate of Lead; Treatment of Chronic Nasal Catarrh—545. Bromide of Potassium; Electro-Cautery for Piles, etc.—546. On the Choice of Anæsthetics; Liquor Picis Alkalinus—547. Muriate of Ammonia; Treatment of Psoriasis—548. Nitrite of Amyl as an Antispasmodic; A New Means of Relieving Toothache; Wash for Falling Hair—549. Nurse's Sore-Mouth; Fluid Extract of Aconite, as a Local Application; Treatment of Pemphigus—550. Electricity as a Means of Resuscitation; Tetanus; Prurigo—551. Gleet Treated with Medicated Bougies; Danger of Performing Operations on the Cervix Uteri; Iron in Rheumatism—552. Treatment of Valvular Diseases of the Heart; Remedy for Influenza; Sweet Tincture of Rhubarb—553. Syphilitic Skin Disease; Tonsilitis; Chronic Rheumatism—534. Loco-motor Ataxia; Cotton-Wool Dressing—555. Tracheotomy by the Galvano-Cautery; Thoracic Aneurism; Hair Restorative—556. The Nature and Treatment of the Constitutional Forms of Eczema; Measles—557. A Remedy for Colds; Galvanism of the Sympathetic in Grave's (Basedow's) Disease; Morfit's Hair Dye—558. Treatment of Varicose Ulcers; Arsenic in Hydrophobia; Gelseminum as an Antiperiodic—559. Variola and Typhus Fever; Infusion of Wild Cherry; Cod-Liver Oil Jelly; Churchill's Syrup of the Hypophosphites—560. Carbolic Acid in Bronchitis; Veratrum Viride in Acute Rheumatism; Diarrhœa Mixture—551. The Temperature in Diphtheria; Local Employment of Chlorate of Potash in Cancerous Sores; Lime Juice and Glycerin—562. Chloroformization During Sleep; Local Treatment of Skin and Syphilitic Diseases; Poisonous Inhalations for Catarrh—563. Treatment of Whooping-cough; Mode of Administering Creosote; Exophthalmic Goitre—564. Post-partum Hemorrhage; Fibrin as a Dietetic; Subacute Pleurisy—565. Local use of Chlorate of Potassa in Open Carcinoma; Treatment of Infantile Cholera; Phosphorus Pills—566. Carbolic Acid in Cerebro-Spinal Meningitis; Disinfectant; Burns—567. Tincture of Iodine; How to Remove Adhesive Plaster; Efficacy of Ice in Gastralgia; Liquor Picis Alkalinus—568. Gelsemium; Horehound in Coughs of Children; Digitalis in the Dropsies and Nephritic Diseases of Children; Fluid to Restore Putrid Tissues; Preservative for Milk; Linimentum Ammoniæ Iodidi; Carbolic Acid in Diphtheritis—569. Camphor Powder in Hospital Gangrene; Muriate of Iron in Erysipelas; Simple Cerate—570. Old French Remedy for Anasarca; Theory and Cure of Diabetes Mellitus—571.

EDITORIAL AND MISCELLANEOUS.

To our Subscribers, . . . . . . . . . . . . . 572
Errata, . . . . . . . . . . . . . . . , . 573
Communications Received; The Address of Drs. Shattuck and Lipscomb, . 574
Dr. M. T. Irby, Corinth, Mississippi; Wood's Household Magazine, . . 575

